# Mental Fatigue and Cognitive Impairment after an Almost Neurological Recovered Stroke

**DOI:** 10.5402/2012/686425

**Published:** 2012-06-25

**Authors:** Birgitta Johansson, Lars Rönnbäck

**Affiliations:** Department of Clinical Neuroscience and Rehabilitation, Institute of Neuroscience and Physiology, The Sahlgrenska Academy, University of Gothenburg, Per Dubbsgatan 14, 1tr, 413 45 Gothenburg, Sweden

## Abstract

Mental fatigue is for many a distressing and long-term problem after stroke. This mental fatigue will make it more difficult for the person to return to work and previous activities. The intention with this study is to investigate mental fatigue in relation to depression and cognitive functions. We examined 24 well-rehabilitated stroke subjects, who suffered from mental fatigue one year or more after a stroke, and 24 healthy controls. Subjects were examined using self-assessment scales for mental fatigue, depression and anxiety, and cognitive tests. The results showed a highly increased rating for mental fatigue for the stroke group (*P* < 0.001). These participants also had a significantly higher rating on the depression (*P* < 0.001) and anxiety (*P* < 0.001) scales. Furthermore, they had a slower information processing speed (*P* < 0.001) and made more errors in a demanding attention and speed test (*P* < 0.05). Among the cognitive tests, processing speed and errors made in an attention and speed test were significant predictors for mental fatigue. We suggest mental fatigue following a stroke to be related to cognitive impairments, primarily information processing speed. Mental fatigue should also be treated as a separate phenomenon and should be differentiated from, and not confused with, depression, even if overlapping symptoms exist.

## 1. Background

Mental fatigue is common and can be a disabling long-time condition following a stroke. It has been estimated that about 30–70% of stroke survivors complain of fatigue [1–7]. Even for those with an almost recovered stroke and without neurological and neuropsychological impairments, mental fatigue can be a distressing problem. The person who suffers from mental fatigue is able to perform mental effort just for short periods, and, notably, it will take longer than normal to regain energy after being exhausted. Accompanying symptoms, such as irritability, sensitivity to stress, concentration difficulties, and emotional instability may further impair social interactions [8–11]. Problems connected to return to work and everyday activities are common. 

Depression after stroke has been the subject of extensive investigation. Studies show that fatigue is sometimes regarded as a component of depression. However, attention has been paid to poststroke fatigue during the last 10 years, and fatigue is now generally held as a separate phenomenon [4–6, 12–14]. Few investigations have been carried out to evaluate fatigue and cognitive functions following a stroke. Leegard reported that fatigue is frequent following a stroke but did not find any related impaired cognitive functions [15]. Van Zandvoort and coauthors investigated lacunar infarct and reported frequent difficulties relating to fatigue and a decreased cognitive performance under more demanding conditions [16].

With the intention to increase knowledge about mental fatigue and cognitive difficulties related to stroke, we examined well-rehabilitated stroke participants, who had suffered from long-term mental fatigue for at least one year prior to examination. We compared the findings for these participants with healthy controls. The subjects were examined for subjective self-reporting of mental fatigue, depression, and anxiety symptoms. Neuropsychological tests were aimed at evaluating information processing speed, attention, and working memory.

## 2. Materials and Methods

### 2.1. Subjects

Twenty-four participants, having recovered from neurological symptoms, but suffering from pathological mental fatigue for at least one year following a stroke and 24 healthy controls were included in the study. The age of the participants was between 30 and 65 years. The study persons were recruited from an advertisement in a local, daily newspaper or from the neurological clinic at the local university hospital and were later included in intervention studies. These studies were approved by the Ethical Review Board, Gothenburg, Sweden. 

The stroke subjects should have been healthy and at work before the stroke meaning that they had no known diseases but hypertonia was present among some participants. The type of stroke was obtained from medical records and self-reports (see Table 1). The pretest data was used in this study for comparison with healthy controls. The control participants were recruited from the local community, with no history of brain injury, stroke, psychiatric or neurological disorder, and no drug abuse, and they were fully able to work. All participants provided an informed consent.

### 2.2. Measures

All participants were assessed for mental fatigue and level of depression and anxiety. They performed cognitive tests focused on information processing speed, attention, and working memory.

#### 2.2.1. Self-Assessment Scales

The self-assessment for mental fatigue is a multidimensional questionnaire containing 15 questions and is adapted from Rödholm et al. [10]. The self-reported questionnaire covers the most common symptoms occurring after brain injury, stroke, or other neurological disorders affecting the brain [11, 17]. The self-assessment scale for mental fatigue and related items has been evaluated, and the 14 questions had adequate internal consistency with a Cronbach's alpha of 0.94. The question relating to 24-hour variation was analysed separately [8, 9]. Each item comprises examples of common activities to be related to four response alternatives. The rating is based on intensity, frequency, and duration. The questions concern fatigue in general, lack of initiative, mental fatigue, mental recovery, concentration difficulties, memory problems, slowness of thinking, sensitivity to stress, increased tendency to become emotional, irritability, sensitivity to light and noise, and decreased or increased duration of sleep as well as 24-hour variations. The construction of the questionnaire resembles the questionnaire from the Comprehensive Psychopathological Rating Scale. This CPRS scale is used here for self-assessment of depression and anxiety [18]. 

#### 2.2.2. Neuropsychological Tests

The neuropsychological tests included Digit Symbol-Coding from the WAIS-III [19], measuring information processing speed, digit span from the WAIS-III, measuring attention and working memory [19]; verbal fluency test, FAS [20]; trail Making Test (TMT) A and B [21], measuring visual scanning, divided attention and motor speed. In order to evaluate higher demands such as dual tasks, a series of two new trail making tests was constructed with three and four factors, respectively [8]. Months were added in part C and both months and days of the week in chronological order in part D. In the latter, the order of letters and digits was switched. Reading speed was measured using a test for screening for dyslexia [22]. A new computer test was constructed in our department including a single and a complex subtest. The single test included speed of mouse click in four squares, located in each corner of a bigger square (6 × 6 cm) on the computer screen and was performed in a clockwise order. A mouse click outside the square was recorded as a miss, and a new click was necessary to do in order to be able to go on with the test. Each session lasted for 30 seconds and was repeated five times. The complex sub-test also included the same mouse clicking procedure, but at the same time, the subject was asked to count how many instances of a specific digit between zero and nine, randomly chosen, he/she could see. The digits were shown on the screen above the mouse click square. Each digit was visible for one second. After the 30 seconds, the subject was asked to report how many of the specific digit he/she had seen. The sub-test was repeated 10 times. It was possible to measure the difference in speed between a single and a complex task, variability over time, and errors made in counting digits. In the complex test, it was necessary to attend to both the square and the digits. 

### 2.3. Statistical Analysis

A comparison between groups was done by *t*-test and analysis of covariance (ANCOVA). The Mann-Whitney *U* test was used when analyzing separate items included in the self-assessment scales. The Bonferroni adjustment was used for multiple comparisons. Pearson's correlation and linear regression were used for analysis of connections between variables. SPSS 16.0 for Windows was used for data analysis.

## 3. Results 

The control group had significantly more years of education than the stroke group (*t*-test, *P* = 0.001), and age was almost significantly different when comparing the two groups (*P* = 0.055, Table 1). Accordingly, ANCOVA, controlling for the variance for education and age, was conducted for all the variables analysed. The only significant gender difference found was that the control females were faster in the Digit Symbol-Coding test, and an ANCOVA, also controlling for sex, was conducted for this variable.

### 3.1. Self-Assessment Questionnaire

**Figure 1:**
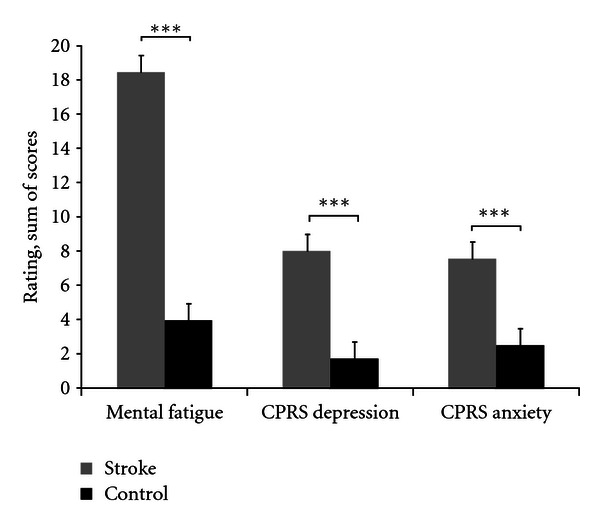
Mean (±SEM) values for the total sum of scores from the self-assessment scales for mental fatigue and the CPRS scale, depression, and anxiety subscale, ***P < 0.001.

A significant difference was found between the groups for the total sum of scores for mental fatigue. This is shown in Table 2. The findings for 24-hour variation are reported separately. The mean value for the stroke group was 18.4 with a 95% confidence interval of between 16.4–20.5. The mean value for the control group was 4.0, with a 95% confidence interval of between 2.9–5.0 (see Figure 1). None of the control subjects reported a value of 10 or above. The total score for the CPRS scale, taken from the depression and anxiety subscale, was also rated significantly higher in the stroke group compared to controls (see Table 2, Figure 1). The significant effect for mental fatigue remained after adjustment for depression (*P* < 0.001).

**Figure 2:**
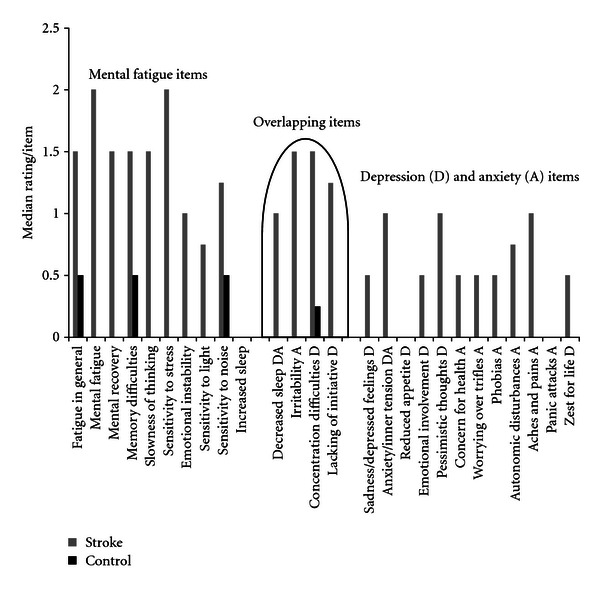
Median values for the separate items from the self-assessment scales for mental fatigue and the CPRS. Median values of zero were common for the items listed for the control group, and also for the item, increased duration of sleep, for the stroke group.

All the separate items in the self-assessment scale for mental fatigue were rated significantly higher for the stroke group compared to controls. The CPRS gave the following findings: without taking into account overlapping items, the items relating to sadness, emotional involvement, pessimistic thoughts, and zest for life rated significantly higher for the stroke group (adjusted for multiple comparisons, as shown in Figure 2). Among the stroke subjects, 74% reported a clear 24-hour variation with morning most frequently reported to be the best time of the day and afternoon and evening the worst. Only 12 % of the control subjects reported a clear 24-hour variation.

### 3.2. Cognitive Tests

**Figure 3:**
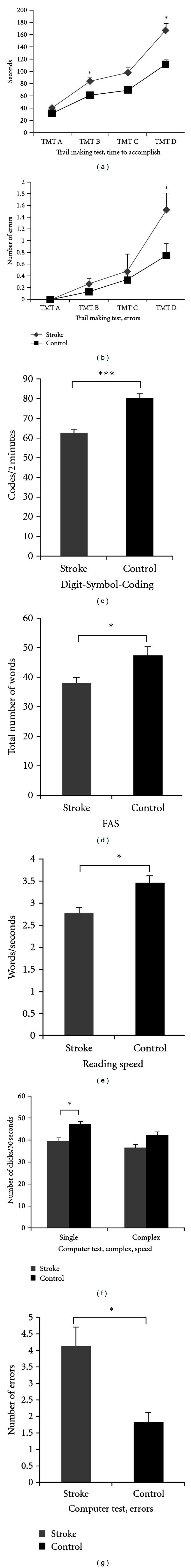
Cognitive tests with significant results, mean ± SEM, *P < 0.05. ***P < 0.001.

The participants from the stroke group were significantly slower on the test measuring information processing speed, primarily Digit Symbol-Coding, and also reading speed and number of mouse clicks in the computer test. The stroke group was also significantly slower and made more errors in TMT D, the most demanding of the TMT tests. They were also significantly slower on TMT B, and produced fewer words on the verbal fluency test. The result for the simple mouse click sub-test was found to be significantly faster for the controls compared with the stroke subjects. The computer test which placed a simultaneous demand on speed, attention, and working memory, resulted in a fairly good speed for the stroke subjects, with no detected difference in speed compared to the controls. On the contrary, the stroke subjects made significantly more errors compared to the controls (Table 2, Figure 3). 

The cognitive tests with significant results (*P* < 0.05, see Table 2) were included in a linear regression model, using the enter method. The model explained 34% of the variance (*R* ^2^  adjusted = 0.337). Digit Symbol-Coding (*P* = 0.004) and the scores for number of errors in the computer test (*P* = 0.018) were significant predictors for mental fatigue, while the other scores were not significant predictors for mental fatigue. Digit Symbol-Coding and number of errors in the computer test also correlated significantly to the mental fatigue sum of scores (*r* = −0.59 and *r* = 0.46).

### 3.3. Mental Fatigue and Depression

The total scores for CPRS, depression, and anxiety were on a significantly higher level compared to controls. The mean level for depression for the stroke group was 8.0. The MADRS, which provides a separate scale, has the same format as the CPRS self-assessment scale depression, except that the items are graded in a different way. In the CPRS scale, depression, the highest level is three for each separate CPRS item, while the levels for the alternatives in the MADRS scale are double for each separate item, with the highest level being 6. According to MADRS, a score between 12 and 20 is regarded as mild depression and 21 and above indicates a probable true depression. The mean level of eight for the stroke subjects corresponds to 16 on MADRS, indicating an overall mild level of depression. 

## 4. Discussion

There was a highly significant difference between the groups on their self-assessment of mental fatigue, with a mean value of 18 reported for the almost recovered stroke group and a mean value of four for the control group. The self-assessment scale has no cut-off value for fatigue, but our experience shows that a value of 15 and above indicates a clear problem with mental fatigue [9, 23]. The clear difference in 24-hour variation also showed the specific exhaustion mental fatigue subjects experience during the active time of the day. The energy they have in the morning will not last for the whole day. It should be noticed that stroke victims were included due to the presence of mental fatigue during one year or more following the stroke. The data we have presented in our study do not include an indication of the frequency of mental fatigue after different types of stroke.

The depression score was significantly higher for the stroke group. However, on an individual basis, according to MADRS, eight participants were not depressed, seven had an indicated mild depression, and nine had a probable, true depression. Among the controls, two participants scored on a mild depression level, while the remainder scored below this level. The total sum of scores on the depression scales may be deceptive if a person is complaining of concentration difficulties and fatigue, but does not have a depressed mood, nor a lack of interest in and enjoyment of daily activities. In this study, three items were overlapping between mental fatigue and depression, and these items were rated on a higher level than the corresponding specific items on the depression sub-scale (see Figure 2). Depression and mental fatigue can occur on their own, but they sometimes occur simultaneously, as shown in this study. Accordingly, we suggest mental fatigue and depression to be independent phenomena following a stroke. This also conforms with the findings from other studies [4–6, 12, 13]. 

The distressing exhaustion along with the bad memory, concentration difficulties, and not being able to perform simultaneous tasks are the phenomena many subjectively complain of following a stroke. It is important to include cognitive tests in the examinations in order to better understand the difficulties connected to mental fatigue and for the purpose of recommending treatment strategies. In this study, physically well-recovered stroke subjects, who had no medical problems except long-term mental fatigue, also showed decreased information processing speed and made more errors in demanding cognitive tasks compared to the control subjects. However, we found that working memory did not deviate from the control subjects. Processing speed is also fundamental and important when considering cognitive functions of a higher order. Few studies have been carried out which cover fatigue and cognitive performance following a stroke. However, studies have shown the connection between fatigue and decreased cognitive performance under more demanding conditions [16], while no cognitive impairments were detected, despite frequent fatigue [15]. With more demanding and sensitive tests, including processing speed and complex attention, it may be possible to detect cognitive impairments accompanying mental fatigue. This is important, as mental fatigue and cognitive deficits will be an obstacle to almost recovered stroke subjects who are on their way to a return to work and previous activities, as every-day life today is complex, with high demands being placed on simultaneous and rapid decisions.

In conclusion, mental fatigue is disabling for many people following an almost recovered stroke, and this is suggested to be related to cognitive impairments, primarily information processing speed, and attention. Mental fatigue should also be treated as a separate phenomenon and should be differentiated from, and not confused with, depression. Today, no specific guidelines exist for the treatment of mental fatigue [24], and research is urgently needed for this common yet distressing symptom. 

## Figures and Tables

**Table 1:** Demographic data for stroke and control participants (mean ± SD).

	Stroke		Controls
Numbers	24		24
Age	54.5 ± 7.0		50.1 ± 3.0
Education in years	14.8 ± 2.8		17.8 ± 4.5
Females/males	13/11		14/10
Time since stroke in years	6.1 ± 7.1		
Percentage of sick leave and numbers	100%/16		
	75%/2		
	50%/2		0%/24
	25%/1		
	0%/3		
Type of stroke and number	Subarachnoid hemorrhage	2	
	Left sided stroke	6	
	Right sided stroke	6	
	Cerebellum stroke	3	
	Brain stem	3	
	Multiple strokes	4	

**Table 2:** Results (ANCOVA) from the variables comparing stroke and control groups, mean ± SD.

Variable	Stroke	Control	*P* value
Mental fatigue scale	18.4 ± 4.9	4.0 ± 2.8	<0.001
CPRS, depression	8.0 ± 3.6	1.7 ± 1.8	<0.001
CPRS, anxiety	7.6 ± 3.8	2.5 ± 2.0	<0.001
TMT A	40.4 ± 15.0	31.0 ± 11.3	0.061
TMT B	83.7 ± 28.4	61.4 ± 15.6	0.031
TMT C	98.3 ± 45.2	69.4 ± 32.0	0.053
TMT D	167.0 ± 58.4	111.0 ± 43.1	0.011
Errors, TMT D^#^	1.5 ± 1.4	0.7 ± 0.9	0.042
Digit Symbol-Coding	62.61 ± 0.6	80.3 ± 11.6	<0.001
Digit span, total	14.3 ± 4.0	16.0 ± 3.7	0.22
Digit span, forward	8.3 ± 2.0	9.2 ± 1.5	0.16
Digit span, backward	6.0 ± 2.2	6.8 ± 2.6	0.80
FAS (total number of words)	38.0 ± 15.1	47.3 ± 1.6	0.035
Reading speed (words/sec)	2.8 ± 0.68	3.5 ± 0.85	0.040
Mouse clicks^∗^	39.4 ± 8.3	47.0 ± 7.8	0.015
Mouse clicks + counting digits^∗^	36.4 ± 8.8	42.3 ± 8.0	0.074
Computer test, errors^∗^	4.1 ± 2.9	1.8 ± 1.7	0.013

^#^Errors on TMT A, B, and C were not significant; ^∗^mean figures for the trials; the first trial was not included.
